# Mesenchymal Stem Cell-Conditioned Media Regulate Steroidogenesis and Inhibit Androgen Secretion in a PCOS Cell Model via BMP-2

**DOI:** 10.3390/ijms22179184

**Published:** 2021-08-25

**Authors:** Rishi Man Chugh, Hang-soo Park, Sahar Esfandyari, Amro Elsharoud, Mara Ulin, Ayman Al-Hendy

**Affiliations:** 1 Department of Surgery, University of Illinois at Chicago, 820 South Wood Street, Chicago, IL 60612, USA; rchugh@kumc.edu (R.M.C.); sesfan2@uic.edu (S.E.); amro.elsharoud@ttuhsc.edu (A.E.); mulin2@uic.edu (M.U.); 2 Department of Radiation Oncology, University of Kansas Medical Center, Kansas City, KS 66160, USA; 3 Department of Obstetrics and Gynecology, University of Chicago, 5841 S. Maryland Ave., Chicago, IL 60637, USA; hspark06@bsd.uchicago.edu

**Keywords:** mesenchymal stem cells, polycystic ovary syndrome, bone morphogenetic protein

## Abstract

Polycystic ovary syndrome (PCOS) is the most common endocrine disorder in women. Previous studies have demonstrated the therapeutic efficacy of human bone marrow mesenchymal stem cells (BM-hMSCs) for PCOS; however, the regulatory mechanism remains unknown. Bone morphogenetic proteins (BMPs) secreted by BM-hMSCs may underlie the therapeutic effect of these cells on PCOS, based on the ability of BMPs to modulate androgen production and alter steroidogenesis pathway enzymes. In this study, we analyze the effect of BMP-2 on androgen production and steroidogenic pathway enzymes in H295R cells as a human PCOS in vitro cell model. In H295R cells, BMP-2 significantly suppressed cell proliferation, androgen production, and expression of androgen-synthesizing genes, as well as inflammatory gene expression. Furthermore, H295R cells treated with the BM-hMSCs secretome in the presence of neutralizing BMP-2 antibody or with BMP-2 gene knockdown showed augmented expression of androgen-producing genes. Taken together, these results indicate that BMP-2 is a key player mediating the favorable effects of the BM-hMSCs secretome in a human PCOS cell model. BMP-2 overexpression could increase the efficacy of BM-hMSC-based therapy, serving as a novel stem cell therapy for patients with intractable PCOS.

## 1. Introduction

Polycystic ovary syndrome (PCOS) is the most common endocrine disorder in women, affecting 4–18% of reproductive-age women [1]. PCOS is characterized by chronic low-grade inflammation and hyperandrogenism caused by excess androgen synthesis by ovarian theca cells [2,3]. Women with PCOS have a greater risk of developing comorbidities later in life, such as type 2 diabetes, cardiovascular diseases, and uterine cancer [4,5,6]. The interaction between ovarian inflammation and altered ovarian androgen synthesis in PCOS may lead to insulin resistance [7,8]. In several published studies, inflammatory cytokines have been shown to stimulate androgen production through upregulation of steroidogenic gene expression, such as CYP11A1, CYP17A1, and 3β-hydroxysteroid dehydrogenase (HSD3β) [9,10,11]. Conversely, inhibition of inflammatory pathways has been shown to reverse PCOS-induced hyperandrogenemia [12].

Previous studies have demonstrated a therapeutic effect of mesenchymal stem cells (MSCs) or their secretome (i.e., conditioned media) in various diseases, including neurological disorders [13], cardiac ischemia [14], diabetes [15], bone and cartilage diseases [16], liver injury or fibrosis [17], myocardial infarction, spinal cord injury, and wound healing [18]. Extensive research over the last decade has focused on the mechanisms driving the immunosuppressive and anti-inflammatory effects of bone marrow mesenchymal stem cells (BM-hMSCs) [19,20]. Cell-to-cell contact and secreted factors are likely to be the main mediators of these effects [21,22]. MSCs have an inherent ability to migrate toward damaged tissues [23,24,25], where they secrete various bioactive mediators, such as growth factors, cytokines, and extracellular vesicles that have immunosuppressive, anti-apoptotic, anti-fibrotic, angiogenic, and anti-inflammatory effects [21,26,27,28]. Recent studies have also demonstrated a therapeutic effect of MSCs on PCOS [29,30]. More recently, we also reported that the BM-hMSCs secretome regulates androgen production in an adrenocortical carcinoma cell line (H295R) similar to ovarian theca cells [31,32,33] and reverses PCOS-related conditions in a letrozole-induced PCOS mouse model [34]. In our previous study, we suggested that IL-10 secreted by BM-hMSCs induces a regulatory pathway for PCOS therapy. However, there could be more therapeutic factors secreted by BM-hMSCs in the secretome, such as cytokines, which may regulate androgen synthesis in PCOS.

Bone morphogenetic proteins (BMPs) are among the many growth factors secreted by BM-hMSCs [35,36]; these proteins play a key role in female fertility [37,38] and are involved in all stages of folliculogenesis. BMPs are multifunctional growth factors that belong to the transforming growth factor β (TGFβ) superfamily [37,39]. Several studies have indicated a decrease in BMP levels in PCOS in both animal models and patients [40,41]. An increasing number of studies suggest that BMPs play an important role in the pathogenesis of PCOS. Theca cells in the ovary proliferate rapidly in PCOS [2,42], and BMP-2 has been shown to inhibit the proliferation of various cells in in vitro culture conditions [43,44,45]. In addition, there is a strong correlation between BMP-2 and BMP-4 and studies have shown that BMP-2, BMP-4, BMP-6, and BMP-7 suppress androgen secretion in bovine theca cells [46]. Together, these studies suggest that BMP-2 may reverse hyperandrogenemia in PCOS.

We hypothesize that BMP-2 secreted by BM-hMSCs drives the observed decrease in androgen production in H295R cells. Here, we show that BMP-2 treatment of H295R cells regulates cell proliferation and reduces cAMP levels and the expression of genes involved in androgen synthesis. We confirmed the effect of BMP-2 in H295R cells by neutralization of BMP-2 in the BM-hMSCs secretome. We also found decreased BMP-2 gene expression in the ovaries of a letrozole-induced PCOS mouse model. Our findings indicate that BMP-2 is a key molecule that regulates theca cell steroidogenesis in PCOS and suggest that BMP-2 may be a candidate molecule for PCOS treatment. Importantly, BMP-2 overexpressing BM-hMSCs could offer a novel stem cell-based therapy for patients with intractable PCOS.

## 2. Results

### 2.1. Effect of BMP-2 on H295R Cell Proliferation and Survival

**Figure 1:**
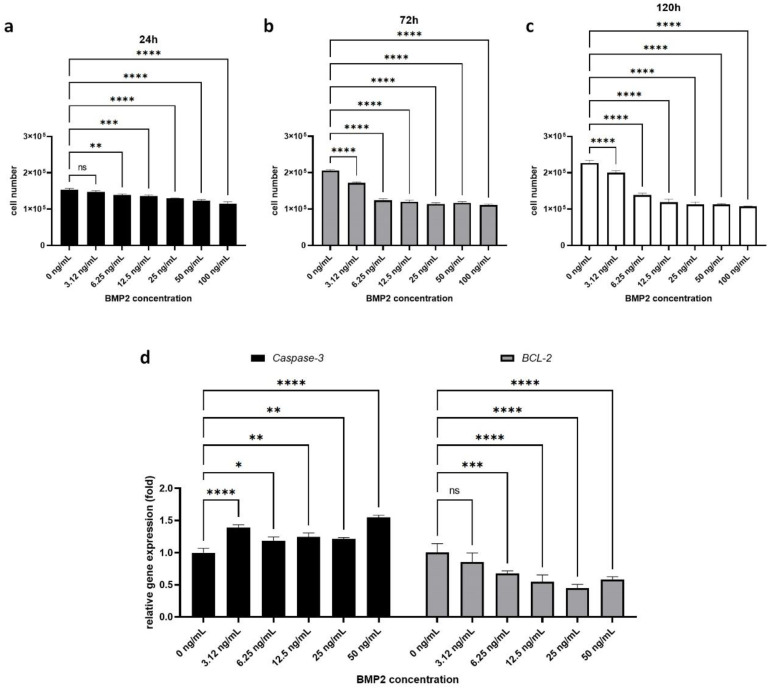
Effect of BMP-2 on H295R cell proliferation. The cell number after 24 h (**a**), 72 h (**b**), and 120 h (**c**) of treatment. (**d**) Relative expression of pro-apoptosis marker gene *Caspase-3* expression, and anti-apoptosis gene *BCL-2* expression. Data are presented as the mean ± SD. (*n* = 3, significance level, * *p* < 0.05, ** *p* < 0.005, *** *p* < 0.0005, **** *p* < 0.0001; ns: Not significant).

Theca cell hyperplasia is a major finding in patients with PCOS and contributes to ovarian androgen oversecretion [2,47,48]; therefore, we evaluated the ability of BMP-2 to inhibit the proliferation of H295R cells in vitro. We found that treatment with human recombinant BMP-2 significantly decreased H295R cell proliferation in a dose- and time-dependent manner (Figure 1a–c). In our cell counting experiment, 24 h after treatment, untreated H295R cells (control) numbered 1.53 ± 0.04 × 10^5^, while other BMP-2-treated cells showed decreased cell numbers (3.125 ng/mL: 1.47 ± 0.04 × 10^5^, 6.25 ng/mL: 1.39 ± 0.03 × 10^5^, 12.5 ng/mL: 1.36 ± 0.03 × 10^5^, 25 ng/mL: 1.29 ± 0.01 × 10^5^, 50 ng/mL: 1.23 ± 0.03 × 10^5^, 100 ng/mL: 1.15 ± 0.06 × 10^5^) compared to untreated H295R cells. After 72 h, BMP-2-treated cells showed significantly decreased cell numbers (3.125 ng/mL: 1.71 ± 0.03 × 10^5^, 6.25 ng/mL: 1.23 ± 0.05 × 10^5^, 12.5 ng/mL: 1.20 ± 0.04 × 10^5^, 25 ng/mL: 1.14 ± 0.03 × 10^5^, 50 ng/mL: 1.17 ± 0.03 × 10^5^, 100 ng/mL: 1.11 ± 0.02 × 10^5^) while untreated control H295R cells were proliferating well (2.05 ± 0.03 × 10^5^). One hundred and twenty hours after treatment, the number of untreated control H295R cells was 2.26 ± 0.08 and that of all of the other BMP-2-treated cells showed a dose-dependently decreased number (3.125 ng/mL: 2.0 ± 0.06 × 10^5^, 6.25 ng/mL: 1.38 ± 0.06 × 10^5^, 12.5 ng/mL: 1.18 ± 0.09 × 10^5^, 25 ng/mL: 1.12 ± 0.07 × 10^5^, 50 ng/mL: 1.12 ± 0.02 × 10^5^, 100 ng/mL: 1.07 ± 0.01 × 10^5^). Furthermore, we found that BMP-2 treatment significantly regulates apoptosis-related gene expression (Figure 1d). Pro-apoptosis marker gene *Caspase-3* expression significantly increased in BMP-2-treated H295R cells (3.125 ng/mL: 1.39 ± 0.04-fold, 6.25 ng/mL: 1.19 ± 0.06-fold, 12.5 ng/mL: 1.25 ± 0.06-fold, 25 ng/mL: 1.22 ± 0.02-fold, 50 ng/mL: 1.55 ± 0.03-fold). On the other hand, anti-apoptosis marker gene *BCL-2* expression was significantly downregulated by BMP-2 treatment (3.125 ng/mL: 0.86 ± 0.14-fold, 6.25 ng/mL: 0.68 ± 0.04-fold, 12.5 ng/mL: 0.55 ± 0.11-fold, 25 ng/mL: 0.45 ± 0.06-fold, 50 ng/mL: 0.58 ± 0.04-fold). Taken together, our results indicate that BMP-2 negatively regulates H295R cell proliferation indirectly by increasing apoptosis.

To further confirm the effect of BMP2 on H295R cell apoptosis at the protein level, we used the Western blot test. We analyzed the protein level of pro-caspase3 and cleaved-caspase3 in BMP2-treated H295R cells (Figure S1). There was no significant difference between control H295R cells and BMP2-treated H295R cells in terms of the pro-caspase level. Unfortunately, cleaved-caspase3 was not detected in our samples. We suggest that the 48 h treatment with BMP2 was sufficient to induce apoptosis at the mRNA level but not enough to observe a significant difference at the protein level.

### 2.2. Effect of BMP-2 on PCOS-Related Parameters in H295R Cells

**Figure 2:**
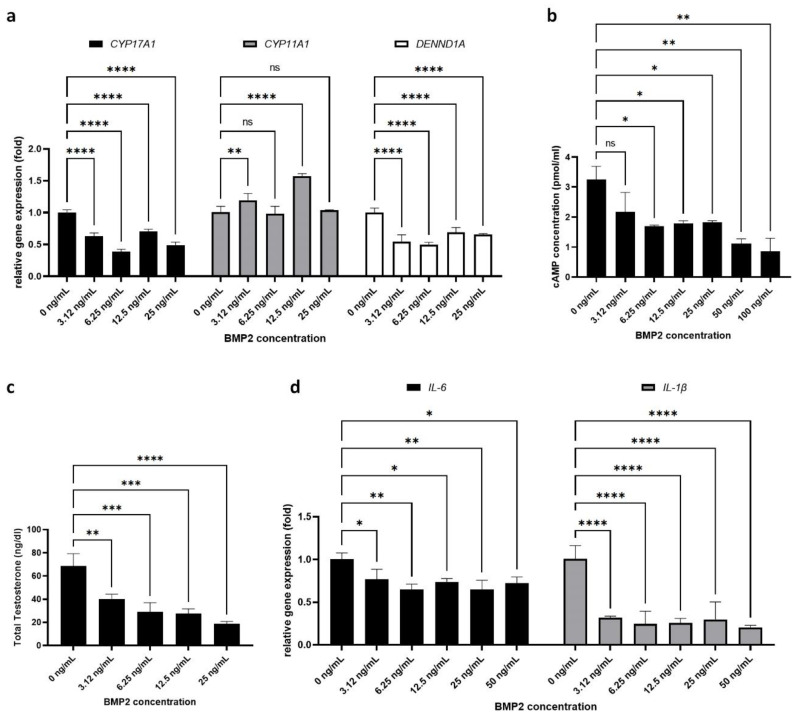
Effect of BMP2 on PCOS-related parameters in H295R cells. (**a**) Relative gene expression of CYP17A1, CYP11A1, and DENND1A in control (0 ng/mL) and BMP2-treated H295R cells (3.125 ng/mL, 6.25 ng/mL, 12.5 ng/mL, 25 ng/mL). (**b**) The cAMP level in H295R cells after treatment with BMP-2 in control (0 ng/mL) and BMP2-treated H295R cells (3.125 ng/mL, 6.25 ng/mL, 12.5 ng/mL, 25 ng/mL, 50 ng/mL, and 100 ng/mL). (**c**) The testosterone level in H295R cells after treatment with BMP-2 in control H295R cells (0 ng/mL) and BMP2-treated H295R cells (3.125 ng/mL, 6.25 ng/mL, 12.5 ng/mL, and 25 ng/mL). (**d**) Relative expression of *IL-6* gene expression and *IL-1β* gene expression in BMP2-treated H295R cells. Data are presented as the mean ± SD. (*n* = 3, significance level, * *p* < 0.05, ** *p* < 0.005, *** *p* < 0.0005, **** *p* < 0.0001; ns: Not significant).

We next assessed the effect of BMP-2 on various PCOS-related parameters in H295R cells. As a first step, we analyzed androgen synthesis by H295R cells. Our RT-PCR results (Figure 2a) show that BMP-2 treatment significantly downregulated the expression of two key androgen-synthesizing enzymes, CYP17A1 (3.125 ng/mL: 0.63 ± 0.04-fold, 6.25 ng/mL: 0.39 ± 0.03-fold, 12.5 ng/mL: 0.70 ± 0.04-fold, 25 ng/mL: 0.49 ± 0.05-fold) and DENND1A (3.125 ng/mL: 0.54 ± 0.11-fold, 6.25 ng/mL: 0.50 ± 0.04-fold, 12.5 ng/mL: 0.69 ± 0.07-fold, 25 ng/mL: 0.66 ± 0.01-fold), but had no significant effect on CYP11A1 gene expression (3.125 ng/mL: 1.19 ± 0.11-fold, 6.25 ng/mL: 0.99 ± 0.11-fold, 12.5 ng/mL: 1.57 ± 0.04-fold, 25 ng/mL: 1.04 ± 0.01-fold).

We also analyzed the protein level of steroidogenesis markers CYP17A1, CYP11A1, and DENND1A by Western blot (Figure S1). Only the DENND1A V1 protein showed decreased expression after BMP2 treatment (0.58 ± 0.20-fold) compared to control H295R cells (1.00 ± 0.17-fold). However, there was no significant difference between control H295R cells and BMP2-treated H295R cells. Similar to our Caspase3 protein results, the 48 h treatment of BMP2 was not sufficient to induce a significant effect on H295R cells at the protein level.

Next, we measured the cAMP levels in H295R cells, as the androgen synthesis cascade requires cAMP (Figure 2b). We found that BMP-2 treatment led to significant suppression of cAMP levels in H295R cells in a dose-dependent manner (control: 3.25 ± 0.44 pmol/mL vs. 3.125 ng/m: 2.18 ± 0.64 pmol/mL, 6.25 ng/mL: 1.69 ± 0.04 pmol/mL, 12.5 ng/mL: 1.79 ± 0.08 pmol/mL, 25 ng/mL: 1.82 ± 0.05 pmol/mL, 50 ng/mL: 1.11 ± 0.16 pmol/mL, 100 ng/mL: 0.87 ± 0.43 pmol/mL).

Based on the negative effect of BMP-2 on both androgen-synthesizing enzyme expression and cAMP levels in H295R cells, we expected that BMP-2 treatment would also lead to decreased levels of androgen secretion. The total testosterone level secreted from control H295R cells (untreated, 0 ng/mL of BMP2) was 68.60 ± 10.67 ng/dL. Indeed, after treatment with BMP-2, the amount of total testosterone in the culture media was significantly decreased in a dose-dependent manner (3.125 ng/mL: 40.17 ± 4.11 ng/dL, 6.25 ng/mL: 28.83 ± 8.2 ng/dL, 12.5 ng/mL: 27.73 ± 3.85 ng/dL, 25 ng/mL: 18.8 ± 1.96 ng/dL). As excess androgen secretion in PCOS is associated with chronic inflammation, we also examined the levels of inflammatory markers in H295R cells after BMP-2 treatment. We found that BMP-2 significantly suppressed the expression of both *IL-6* (3.125 ng/mL: 0.76 ± 0.12-fold, 6.25 ng/mL: 0.65 ± 0.06-fold, 12.5 ng/mL: 0.73 ± 0.04-fold, 25 ng/mL: 0.64 ± 0.11-fold, 50 ng/mL: 0.72 ± 0.07-fold) and *IL-1b* (3.125 ng/mL: 0.32 ± 0.02-fold, 6.25 ng/mL: 0.25 ± 0.15-fold, 12.5 ng/mL: 0.26 ± 0.05-fold, 25 ng/mL: 0.30 ± 0.20-fold, 50 ng/mL: 0.20 ± 0.03-fold) at all tested concentrations (Figure 2d). These results support a link between androgen excess and inflammation in PCOS, and suggest that therapies that control androgen secretion may also reduce inflammation.

### 2.3. Estimation of BMP-2 Secretion by BM-hMSCs

**Figure 3:**
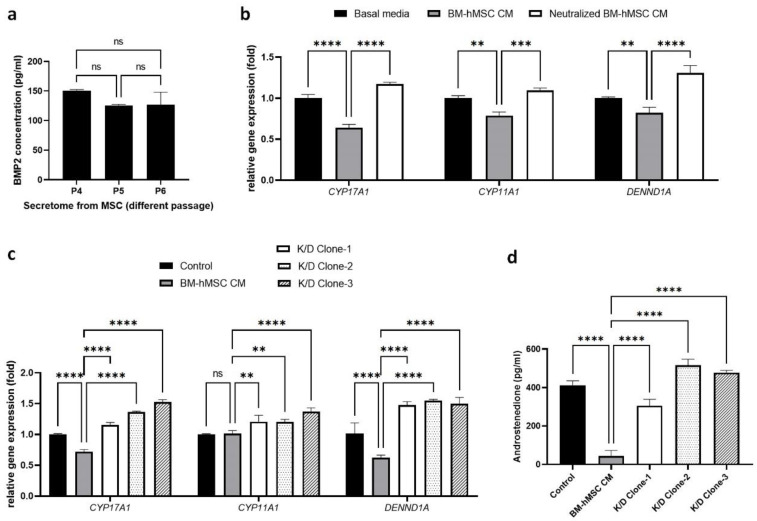
BMP-2 secretion in BM-hMSCs. (**a**) BMP-2 concentration in different passages of BM-hMSCs, P3: 150.75 ± 1.76, P4: 124.5 ± 1.78, P5: 127.0 ± 21.21. (**b**) Effect of neutralized secretome on androgen synthesizing genes’ (CYP17A1, CYP11A1, and DENND1A) expression. Relative gene expression in Control H295R cells (basal media), MSC conditioned media-treated H295R cells (BM-hMSC CM), and BMP2 neutralized conditioned media-treated H295R cells (Neutralized CM). (**c**) Effect of BMP-2 knockdown MSCs clone secretome on androgen-synthesizing genes’ (CYP17A1, CYP11A1, and DENND1A) expression. (**d**) Effect of BMP-2 knockdown MSCs clone secretome on androstenedione synthesis. Data are presented as the mean ± SD. (*n* = 3, significance level, ** *p* < 0.005, *** *p* < 0.0005, **** *p* < 0.0001; ns: Not significant.)

Our previous studies revealed that the BM-hMSCs secretome regulates steroidogenesis pathway genes in H295R cells. To identify the factors in the BM-hMSCs secretome that mediate this effect, we first performed a literature search. BMP-2 emerged as a candidate factor; it is released by BM-hMSCs [49] and has been shown in various studies to control ovarian androgen levels in bovine theca cells [50]. We quantified the concentration of BMP-2 in the BM-hMSCs secretome by ELISA, using passages P3 to P5 BM-hMSCs (Figure 3a). BMP-2 secretion was highest in P3 BM-hMSCs (150.75 ± 1.76 pg/mL) but was not significant between P4 (124.5 ± 1.78 pg/mL) and P5 (127.0 ± 21.21 pg/mL). In the following experiments, we used the P3 BM-hMSCs secretome for the treatment of H295R cells to evaluate the effect of BMP2 present in the MSC secretome.

To confirm that BMP-2 in the BM-hMSCs secretome regulates steroidogenesis in H295R cells, we neutralized BMP-2 in the BM-hMSCs secretome by an antibody-based neutralizing method. We use the neutralized secretome to treat H295R cells and analyze the expression of steroidogenesis pathway genes (Figure 3b). In our result, BM-MSCs secretome treatment of H295R cells led to decreased expression of CYP17A1 (0.64 ± 0.04-fold), CYP11A1 (0.78 ± 0.04-fold), and DENND1A (0.82 ± 0.06-fold) gene expression compared to control H295R cells. Interestingly, in the BMP-2-neutralized BM-hMSCs secretome-treated group, H295R cells showed a significant upregulation of androgen-synthesizing genes CYP17A1 (1.17 ± 0.02-fold), CYP11A1 (1.09 ± 0.03-fold), and DENND1A (1.30 ± 0.10-fold).

To further confirm the effect of BMP-2 in the BM-hMSCs secretome, we knocked down BMP-2 at the gene level using lentiviral particles followed by puromycin C selection. The secretome collected from BMP-2 knockdown BM-hMSCs (K/D clone) was used to treat H295R cells and measure the effect on androgen-synthesizing gene expression (Figure 3c). Our results show that BMP-2 knockdown nullified the suppression effect of MSC conditioned media on androgen-synthesizing genes. We found that K/D clone secretome treatment significantly upregulated the androgen-synthesizing genes CYP17A1 (Clone 1 CM: 1.16 ± 0.04-fold, Clone 2 CM: 1.36 ± 0.01-fold, Clone 3 CM: 1.52 ± 0.04-fold) and DENND1A (Clone 1 CM: 1.47 ± 0.05-fold, Clone 2 CM: 1.55 ± 0.02-fold, Clone 3 CM: 1.50 ± 0.10-fold) in H295R cells compared to the wild-type BM-hMSCs secretome. CYP11A1 expression also increased in K/D clone CM-treated H295R cells (Clone 1 CM: 1.21 ± 0.10-fold, Clone 2 CM: 1.21 ± 0.04-fold, Clone 3 CM: 1.37 ± 0.06-fold), while there were no significant changes in the wild-type BM-hMSCs secretome-treated H295R cells (1.01 ± 0.05-fold). We also detected significantly higher levels of secreted androgen from H295R cells after treatment with the K/D clone CM (Clone 1 CM: 0.31 ± 0.03-fold, Clone 2 CM: 0.52 ± 0.03-fold, Clone 3 CM: 0.48 ± 0.01-fold) compared to the cells treated with the wild-type BM-hMSCs secretome (0.04 ± 0.02-fold) (Figure 3d). Together, these data confirm the role of BMP-2 as the component of the BM-hMSC secretome that regulates androgen synthesis in H295R cells as an in vitro PCOS cell model.

### 2.4. Effect of BM-hMSCs on Ovarian Tissue

**Figure 4:**
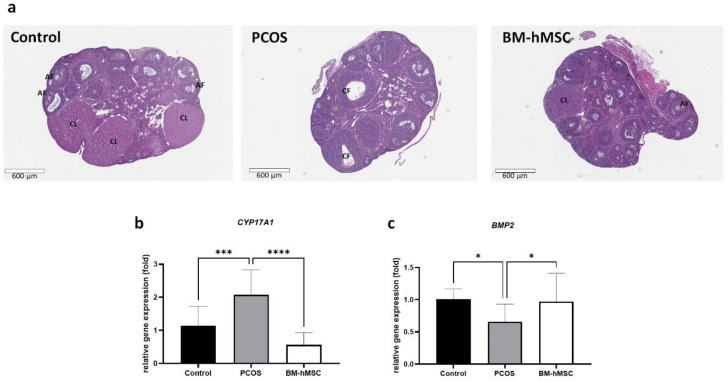
Effect of hBM-MSCs on ovarian tissue. (**a**) Morphology of ovary (H & E staining) in healthy mouse (control), PCOS mouse (PCOS), and BM-hMSC-treated mouse (BM-hMSCs). (**b**) Relative expression of CYP17A1 gene in healthy mouse (control), PCOS mouse (PCOS), and BM-hMSC-treated mouse (BM-hMSCs). (**c**) Relative expression of BMP-2 gene in healthy mouse (control), PCOS mouse (PCOS), and BM-hMSC-treated mouse (hBM-MSCs). Data are presented as the mean ± SD. (Number of animals = 6/group, significance level, * *p* < 0.05, *** *p* < 0.0005, **** *p* < 0.0001).

Next, we examined the therapeutic effect of BM-hMSCs in a letrozole-induced PCOS mouse model established in a previous study [51]. We injected 5 × 10^5^ BM-hMSCs per ovary and collected the ovarian tissue for histological analysis after two weeks of cell implantation. Histological analysis using H & E staining revealed numerous mature follicles and corpora lutea, which represent normal ovulation, in control ovaries, while PCOS ovaries showed a lower number of antral follicles, no corpora lutea, and evidence of multiple cysts. In BM-hMSC-treated ovaries, we observed a partial restoration of corpora lutea and antral follicles, which appeared morphologically similar to those in the healthy control group (Figure 4a).

Next, we analyzed gene expression in the ovary tissue by quantitative RT-PCR. In PCOS mouse ovaries, CYP17A1 gene expression was significantly increased (2.08 ± 0.48-fold) compared to healthy control mouse ovaries (Figure 4b). In contrast, CYP17A1 gene expression in BM-hMSC-treated ovaries was significantly decreased (0.57 ± 0.23-fold) compared to untreated PCOS mouse ovaries, confirming the ability of hBM-MSCs to reverse the PCOS characteristics in this mouse model. Next, we analyzed BMP2 gene expression in the mouse ovaries (Figure 4c). We found decreased BMP-2 gene expression in the PCOS mouse ovary (0.66 ± 0.18-fold), which was restored after BM-hMSC treatment (0.97 ± 0.28-fold). Thus, the restoration of ovulation and normal androgen steroidogenic gene expression levels in the ovary after BM-hMSC engraftment in the letrozole-induced PCOS animal model was associated with an increase in ovarian BMP-2 expression.

## 3. Discussion

In this study, we found that BMP-2 inhibits cell proliferation and apoptosis gene expression in the H295R cell line as an in vitro model of PCOS. Moreover, BMP-2 treatment suppressed androgen secretion by decreasing the gene expression of CYP17A1 and DENND1A. We found that BMP-2 is secreted by BM-hMSCs and that depletion of BMP-2 from the BM-hMSC secretome reverses the therapeutic effect of the secretome on reducing androgen production and inflammatory marker expression. Our in vivo data further suggest that intraovarian injection of BM-hMSC stimulates BMP2 gene expression and suppresses androgen-synthesizing gene CYP17A1 expression in the PCOS mouse ovary.

H295R cells are well-known androgen-producing cells used in many published studies [31,52,53]. Regulating androgen production is an important aspect of PCOS treatment because approximately half of PCOS patients show excessive adrenal androgen production [31,52,53]. Suppression of androgen-producing cells could thus be a main strategy to treat PCOS. In this study, we chose the dose range of BMP-2 on the basis of a previous study [43,44,45]. Our data showed that BMP-2 successfully inhibited H295R cell proliferation by stimulating apoptosis. BMP-2 also suppressed androgen production through the inhibition of androgen synthesis enzyme CYP17A1 and DENND1A gene expression. Taken together, our data demonstrate that BMP-2 is a promising molecule for PCOS treatment.

In this study, we reported the therapeutic benefits of BMP2. However, BMP2 is known to induce osteogenesis and chondrogenesis in MSC [54,55]. Due to the high osteogenic and chondrogenic potency of MSC, overexpression of BMP2 may lead to unexpected differentiation of MSC. In the case of PCOS, the intraovarian injection of MSCs, forming osteocytes or chondrocytes in the ovary, is not an ideal situation. Fortunately, endogenous expression of BMP2 did not affect their characteristics. Moreover, our study showed that a very low concentration of BMP2 (6.25 ng/mL), and even endogenous secretion by MSC (124.5 ± 1.78 pg/mL) could suppress androgen production in H295R cells.

In a previous study, 100 ng/mL of BMP2 was used to stimulate osteogenic or chondrogenic differentiation in MSC [56,57]. Taken together, the low concentration of BMP2 used in our study is sufficient to suppress androgen production and may not induce osteogenic and chondrogenic differentiation in engrafted MSCs. In addition, we did not find any osteogenic or chondrogenic structures in our mice ovarian samples. To nullify the concerns about side effects, further in vitro and in vivo assays are needed on BMP2 overexpression to help us understand the safety issues and side effects of high-level BMP2 in MSCs.

Previous studies have reported the therapeutic potential of MSCs in various conditions such as acute lung injury (ALI), asthma, primary ovarian insufficiency (POI), and PCOS [22,58,59,60,61]. MSCs are considered an ideal cell source for cell-based therapy due to their immune suppressive potential [20]. Further studies reported that the immunosuppressive potential of MSCs led to suppressed inflammation and delayed or prevented allorejection of transplanted MSCs [62], which suggests that the immunoregulatory properties of MSCs make them an ideal cell therapeutic candidate [19]. The therapeutic effect of MSC not only relies on their differentiation property but on various secreting factors, such as cytokines and extracellular vesicles [21,22,63]. These secreted cytokines or therapeutic factors can regulate the abnormal status of the target cells. Based on these studies, we also reported that MSCs can be considered a bio-organ that may treat female infertility disorders [58]. Taken together, MSCs are a promising candidate as a bio-platform that secretes various therapeutic factors and could be novel options for future cell-based therapies.

In this study, we targeted PCOS, which shows hyperandrogenemia [64]. Based on previous studies, higher androgen production in women may lead to other major symptoms of PSOC such as inflammation and metabolic disorder [9,12,65]. In a previous study, we also demonstrated a positive feedback loop, the so-called loop of hyperandrogenemia [34], between major parameters of PCOS. It is important to regulate androgen production by androgen-producing cells, which could eventually restore fertility in PCOS patients.

We previously reported that BM-hMSCs secrete BMP-2. In our animal experiments, we injected hBM-MSCs cells as a source of BMP-2 into the ovaries of a PCOS mouse model. As expected, we found that BM-hMSCs treatment inhibited CYP17A1 gene expression in PCOS mouse ovaries compared to untreated PCOS mouse ovaries. Interestingly, BM-hMSC treatment also enhanced BMP-2 gene expression in mouse ovary tissue. This suggests that BM-hMSCs not only secrete BMP-2 but also stimulate BMP-2 gene expression in mouse ovarian tissue.

Although we used human cells for our in vitro experiments, human and mouse BMP-2 share 92% amino acid identity [66], which may permit cross-species reactivity [67]. Previous studies reported a high probability of cytokine cross-reactivity between humans and mice at above 80% amino acid identity [66]. Therefore, it is not surprising that human BMP-2 secreted from injected BM-hMSCs could suppress androgen production and stimulate ovarian BMP-2 gene expression in a PCOS mouse model.

Our data suggest a therapeutic effect of BM-hMSC-derived BMP-2 in PCOS. MSCs secrete various factors in addition to BMP-2, including cytokines and exosomes, which can regulate cell metabolism and gene expression. Although we reported the effect of BMP-2, it may be possible that some other factors secreted by BM-hMSCs underlie their therapeutic effects in PCOS—namely, inhibition of theca cell proliferation, androgen production, and inflammatory markers. Identifying the protein profile or exosomal RNA profile of the BM-hMSC secretome might be useful to find more promising therapeutic factors to control female reproductive diseases. It is important to understand the therapeutic mechanism of BM-hMSC for further studies.

The proteomic profile of BM-hMSC secretome could help us to develop a novel approach to the treatment of PCOS. For example, it may be possible to enhance the therapeutic effect of BM-hMSCs in PCOS through overexpression of BMP-2 to downregulate ovarian androgen production. We found that the concentration of BMP-2 in the BM-hMSC secretome was much lower than in our in vitro study using recombinant BMP-2. Moreover, we reported a dose-dependent effect of BMP-2 in H295R cells for the steroidogenesis pathway. We can therefore expect a higher therapeutic effect if BM-hMSCs secrete more BMP-2. MSCs engineered to overexpress these secreted factors could be a promising future treatment option for women with intractable PCOS.

Although we reported a therapeutic effect of MSC on PCOS condition through BMP2, our study still has some limitations. Our Western blot results (Supplementary Figure S1) did not show a significant difference between control and BMP2-treated H295R cells due to the high sample-to-sample variation. In this study, we administered BMP2 to H295R cells for 48 h. The 48 h treatment was sufficient to analyze mRNA level changes but not protein-level changes. Analyzing the long-term effect of BMP2 in PCOS with more samples might be an interesting topic for future study. Using MSCs from only one donor is another limitation of this study. Other MSCs isolated from different donors may show different BMP2 expression patterns. In addition, a recent study also shows that, even in the same bone marrow, MSCs show cell-to-cell variation in terms of their cytokine production [68]. To apply these findings in a clinical setting, it is important to confirm the reproducibility of the findings using multiple donor MSCs. Our in vivo data only showed morphology and mRNA expression in the ovary tissue. Due to the small size of tissue (mouse ovary), we used the whole ovary to isolate enough mRNA to check the expression of the genes. Moreover, to overcome animal-to-animal variation, we had to use all animals for RT-PCR assay. Further studies with a large sample size are needed to perform the ovarian gene expression experiment not only at the RNA level but also at the protein level.

Our study shows that the BMP-2 secreted by BM-hMSC can treat hyperandrogenemia by suppressing steroidogenesis and gene expression. Therapy using BMP-2 may represent a novel therapeutic option for women with PCOS.

## 4. Materials and Methods

### 4.1. Human Mesenchymal Stem Cell Culture

BM-hMSCs were purchased from Roosterbio (Frederick, MD, USA). These cells were originally isolated from the bone marrow of a 29-year-old female donor. For BM-hMSCs culture, around 3000 cells/cm^2^ were plated in a Corning CellBIND^®^ T75 cell culture flask (Corning, NY, USA). BM-hMSCs were cultured with the recommended cell culture medium, RoosterNourish™-MSC-XF (Roosterbio, Frederick, MD, USA), per the expansion protocol. When the culture reached approximately 80% confluence, cells were trypsinized using CTSTM TrypLE select enzyme (Gibco, Waltham, MA, USA) and serially expanded for two more passages before use in experiments. Parallel flasks were used for the collection of conditioned media (BM-hMSCs secretome). All cultured cells in this study were tested for mycoplasma using a MycoAlert^TM^ mycoplasma detection kit (Lonza, Basel, Switzerland), and all cells cultured were free from mycoplasma contamination.

### 4.2. Human Adrenocortical Carcinoma Cell Line (H295R) Culture

H295R cells were used as an in vitro cell culture model of PCOS. H295R cells were purchased from ATCC (Manassas, VA, USA) and cultured per the recommended protocol. Briefly, H295R cells were cultured in flasks precoated with extracellular matrix (Gibco, Waltham, MA, USA) with DMEM/F12 (Gibco, Waltham, MA, USA) and 2.5% Nu-Serum (Corning, NY, USA). Cells were subcultured at a ratio of 1:3 to 1:4 and the culture medium was changed twice a week.

### 4.3. Collection of Secretome from BM-hMSCs

Conditioned media were prepared from passage three to five BM-hMSCs. When BM-MSCs cultures reached approximately 80–90% confluence, the medium was collected from the flask and discarded. Cells were cultured with RoosterBasal™-MSC growth supplement free (Roosterbio) for 24 h and then conditioned media were collected from the flasks, centrifuged at 500× *g* for 10 min at 4 °C to remove the cell debris, aliquoted and stored at –80 °C for further use. RoosterBasal™-MSC basal media without cells were also incubated for 24 h in a culture flask for use as a negative control.

### 4.4. Treatment of H295R Cells with BMP-2

H295R cells were cultured on six-well plates precoated with an extracellular matrix at a density of 18 × 10^4^ cells per well and cultured for 48 h. Cells were treated with 0 to 50 ng/mL recombinant human BMP-2 (R & D Systems, Minneapolis, MN, USA) in H295R culture media for 48 h. These concentrations were chosen based on a previous study using bovine theca cells [69]. After removal of treatment media, the cells were washed with PBS three times before adding basal media (serum-free) and incubating for another 24 h. To compare the cell number, cells was determined using a CTSTM TrypLE select enzyme (Gibco, Waltham, MA, USA). The number of live cells was counted by Trypan blue assay, which is the most widely used and still the gold standard method to perform cell viability assays in cell culture [70,71]. Cells were collected to analyze the expression of steroidogenesis pathway genes. The cell culture supernatant was used for chemiluminescent quantification of testosterone released by H295R cells using an automated UniCel DxI 800 Access Immunoassay System (Beckman Coulter, Inc., Brea, CA, USA) [72].

### 4.5. Effect of BMP-2 on H295R Cells

After 48 h of treatment with different concentrations of recombinant human BMP-2, H295R cell proliferation was monitored for an additional 24, 72, and 120 h. Markers of cell apoptosis (*Caspase-3*), anti-apoptosis (*Bcl-2*), and inflammation (*IL-6* and *IL-1β*) were examined by quantitative RT-PCR after 48 h of BMP-2 treatment. The cAMP level in the conditioned media was measured by ELISA (Abcam, Cambridge, MA, USA) after 48 h of BMP-2 treatment.

### 4.6. ELISA for BMP-2

To investigate the levels of BMP-2 secreted by BM-hMSCs, we measured the amount of BMP-2 in the culture media of BM-hMSCs at passages three to five by ELISA, following the manufacturer’s instructions (CAT no. RAB0028, Millipore, Burlington, MA, USA). The detection limit of the ELISA kit was 45 pg/mL, while the intra- and interassay coefficients of variation (CV) were <10% and <12%, respectively.

### 4.7. Neutralization of BMP-2 in Conditioned Media

To confirm the role of BMP-2 in the regulation of steroidogenic gene expression in H295R cells, the BM-hMSCs secretome was incubated in six-well plates previously coated with a neutralizing antibody against human BMP-2 (Abcam, Cambridge, MA, USA) for 2 h at 37 °C and under sterile conditions. After incubation, the secretome was collected and applied to H295R cells. After treatment, cells were trypsinized and collected for quantitative RT-PCR.

### 4.8. Knockdown of BMP-2 in Mesenchymal Stem Cells

BM-hMSCs were seeded onto six-well plates at a density of 8 × 10^4^ cells per well and treated with human BMP-2 shRNA lentivirus (OriGene Technologies, Rockville, MD, USA) (MOI 50) at 8 µg/mL of polybrene per the manufacturer’s protocol. After lentivirus treatment, BM-MSCs were plated at a 1:10 ratio for the selection of puromycin C-resistant colonies. Three different colonies of BM-MSCs with BMP-2 knockdown were selected and expanded for the collection of conditioned media to treat H295R cells.

### 4.9. Quantitative RT-PCR

RNA isolation was performed using a TRIzol Reagent (Invitrogen, Waltham, MA, USA) according to the manufacturer’s instructions. The concentration and purity of RNA were quantified by spectrophotometry at 260 nm using Nanodrop 2000 (Thermo Fisher Scientific, Waltham, MA, USA). One microgram of total RNA was reverse-transcribed using the RNA to cDNA EcoDry premix (Takara Bio, Kusatsu, Japan). Real-time PCR was performed using the CFX96 PCR instrument with matched primers (Table 1) and Universal SYBR Green Supermix (Bio-Rad, Hercules, CA, USA). The following PCR parameters were used: initial denaturation cycle at 95 °C for 3 min, followed by 40 amplification cycles at 95 °C for 10 s, 56 °C for 15 s, and 72 °C for 1 min. The results are presented as the fold change in relative gene expression quantified using the delta–delta CT (ΔΔCt) method. Beta-actin was used as a reference gene for sample normalization [34,73,74].

### 4.10. Western Blot Analysis

Control H295R cells and BMP-2-treated (25 ng/mL, 48 h) H295R cells were lysed with a RIPA buffer (Cell Signaling, Danvers, MA, USA) containing protease and a phosphatase inhibitor cocktail (Thermo Fisher Scientific, Waltham, MA, USA). The protein preparation and SDS-PAGE electrophoresis were performed through the protocol used in our previous study [34]. After the protein was transferred onto the PVDF membrane (Bio-Rad, Hercules, CA, USA), blocked membranes were incubated in 5% nonfat dry milk in 1× PBS overnight at 4 °C with primary antibodies against CYP17A1 (ab125022, 1:500 dilution, Abcam, Cambridge, MA, USA), CYP11A1 (ab75497, 1:500 dilution, Abcam), DENND1A (LS-C167356, 1:250, LSBio), and β-actin (clone AC-15, A5441, 1:5000, Sigma) in 1% nonfat dry milk in 1x PBS overnight at 4 °C. Caspase3 was analyzed using the apoptosis Western blot cocktail antibody (ab136812, Abcam) as per the manufacturer’s recommended dilution conditions. After washing, the membrane was incubated with the appropriate HRP-linked secondary antibodies (7076 and 7074 Cell Signaling; ab136812, Abcam) in 1% nonfat dry milk in 1x PBS at room temperature for 1 h. The membrane was developed and visualized with Trident Femto Western HRP substrate (GeneTex, Irvine, CA, USA) and ChemiDoc XRS + molecular imager (Bio-Rad). After imaging, the membranes were stripped with a RestoreTM PLUS stripping buffer (Thermo Scientific, Waltham, MA, USA) to incubate with another antibody. The signal density of each protein band was quantified using ImageJ software (U.S. National Institutes of Health, Bethesda, MD, USA) and normalized against the corresponding β-actin band.

### 4.11. PCOS Mouse Model and Intra-Ovarian Injection of BM-hMSC

Animal experiments in this study were approved by the University of Illinois at Chicago Animal Care Committee (UIC ACC). All animal experiments were performed in compliance with the University of Illinois at Chicago’s policies and guidelines for use of laboratory animals. At four weeks of age, mice (Charles River, Wilmington, MA; *n* = 6/group) were subcutaneously implanted with a placebo or 5 mg LTZ pellet (Innovative Research of America, Sarasota, FL, USA), which provides a constant release of LTZ (50 μg/day). Body weight was monitored weekly post-implantation to assess PCOS-related obesity. Five weeks after LTZ pellet implantation, mice underwent intra-ovarian injection of BM-hMSCs (5 × 10^5^ cells/ovary) via laparotomy. For the control group and untreated PCOS group, 10 µL of PBS was injected into the ovaries. Two weeks after BM-hMSCs’ engraftment, mice were anesthetized and ovaries were collected for further analysis.

### 4.12. Statistical Analysis

Comparisons between groups were made by two-way ANOVA using GraphPad Prism 9 (GraphPad Software, San Diego, CA, USA). A difference between groups of *p* < 0.05 was considered significant.

## Figures and Tables

**Table 1:** List of primers.

Gene	Forward Primer (5′–3′)	Reverse Primer (5′–3′)
Human *ACTB*	TGGATCAGCAAGCAGGAGTATG	GCATTTGCGGTGGACGAT
Human *CYP17A1*	GGCCTCAAATGGCAACTCTAGA	CTTCTGATCGCCATCCTTGAA
Human *CYP11A1*	GAGGGAGACGGGCACACA	TGACATAAACCGACTCCACGTT
Human *DENND1A*	CAATTCCCGGAGGACTACAGT	AGCACGAATGTGAAGTTCTGG
Human *IL6*	TGCACTTTATGACGCACTCAC	TGTCCAAAAACACGAAATCATGC
Human *IL1B*	ATGATGGCTTATTACAGTGGCAA	GTCGGAGATTCGTAGCTGGA
Human *BCL2*	AACGTGCCTCATGAAATAAG	TTATTGGATGTGCTTTGCATTC
Human *CASP3*	TGTTTGTGTGCTTCTGAGCC	CACGCCATGTCATCATCAAC
Mouse *Gapdh*	CACATTGGGGGTAGGAACAC	AACTTTGGCATTGTGGAAGG
Mouse *Cyp17a1*	GAGTTTGCCATCCCGAAGGA	CCAGCTCCGAAGGGCAAATA
Mouse *Bmp2*	TAGATCTGTACCGCAGGCA	CCGTTTTCCCACTCATCTCT

## Data Availability

Data is contained within the article and Supplementary Material.

## Supplementary Materials

The supplementary materials are available online at https://www.mdpi.com/article/10.3390/ijms22179184/s1.

## Abbreviations

BM-hMSC	Bone marrow-derived human mesenchymal stem cell
PCOS	Polycystic ovary syndrome
BMP2	Bone morphogenetic proteins 2
ELISA	Enzyme-linked immunosorbent assay
qRT-PCR	Quantitative real-time polymerase chain reaction
IHC	Immunohistochemistry
IL-1β	Interleukin-1 beta
IL-6	Interleukin-6
MSC CM	Mesenchymal stem cell conditioned medium (secretome)
FBS	Fetal bovine serum
PBS	Phosphate-buffered saline
UIC ACC	University of Illinois at Chicago Animal Care Committee
LTZ	Letrozole
H & E	Hematoxylin–eosin
