# Supplementary material for: Mesenchymal Stem Cell-Conditioned Media Regulate Steroidogenesis and Inhibit Androgen Secretion in a PCOS Cell Model via BMP-2
Source: Int J Mol Sci. 2021 Aug 25;22(17):9184. doi: 10.3390/ijms22179184 (PMC8431467; doi:10.3390/ijms22179184)
Supplement: Supplementary file 1 [file ijms-22-09184-s001.zip › ijms-1308334-supplementary.pdf]

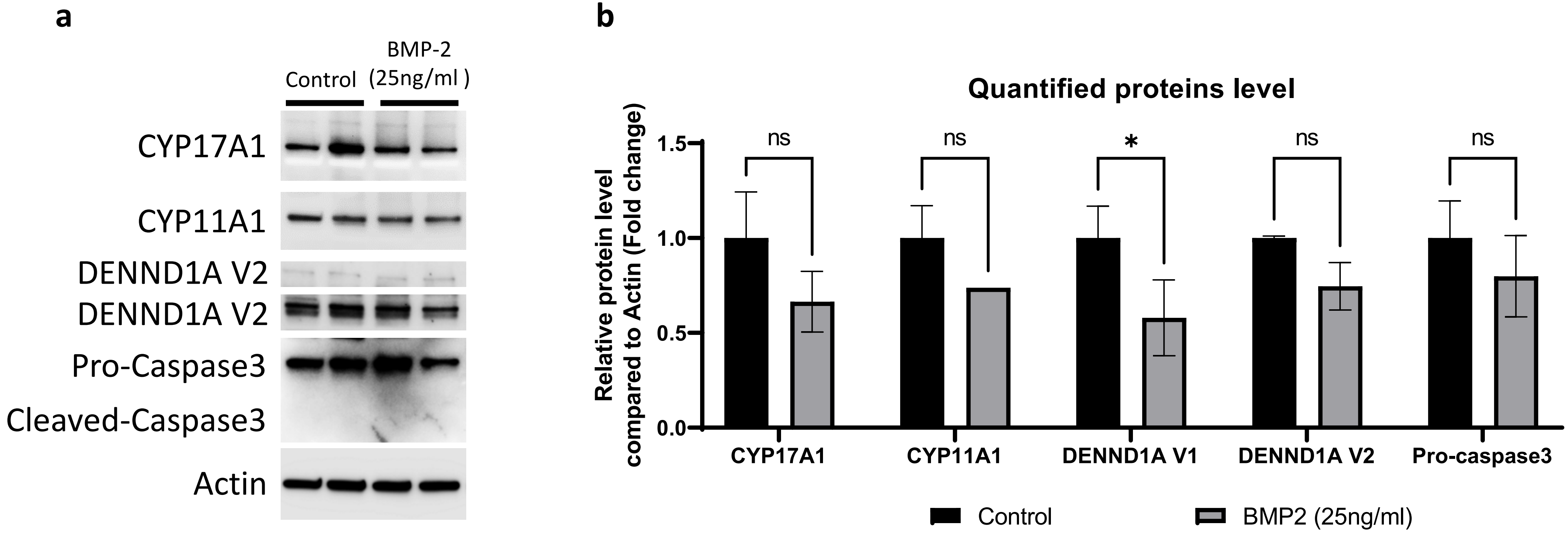

Supplementary Figure S1. Protein expression changes in H295R cells after BMP-2 treatment.
(a) Western blot result with CYP17A1, CYP11A1 , DENND1A varient1(DENND1A V1), DENND1A varient1(DENND1A V1) and Caspase3 in control H295R cells and BMP-2 treated (25ng/ml) H235R cells. The cleaved Caspase3 were not detected in this samples. (b) Relative protein level of CYP17A1, CYP11A1 , DENND1A varient1(DENND1A V1), DENND1A varient1(DENND1A V1) and Pro-Caspase3. Data presented as the mean ± SD. (n=2, Significant level, *: $p<0.05$; NS: Not significant).
